# Antimicrobial Prophylaxis for Urologic Procedures in Paediatric Patients: A RAND/UCLA Appropriateness Method Consensus Study in Italy

**DOI:** 10.3390/antibiotics11030296

**Published:** 2022-02-23

**Authors:** Susanna Esposito, Erika Rigotti, Alberto Argentiero, Caterina Caminiti, Elio Castagnola, Laura Lancella, Elisabetta Venturini, Maia De Luca, Stefania La Grutta, Mario Lima, Simonetta Tesoro, Matilde Ciccia, Annamaria Staiano, Giovanni Autore, Giorgio Piacentini, Nicola Principi, The Peri-Operative Prophylaxis in Neonatal and Paediatric Age (POP-NeoPed) Study Group

**Affiliations:** 1 Pediatric Clinic, University Hospital, Department of Medicine and Surgery, University of Parma, 43126 Parma, Italy; alberto.argentiero@unipr.it (A.A.); giovanniautore@gmail.com (G.A.); 2 Pediatric Unit, Department of Surgical Sciences, Dentistry, Gynecology and Pediatrics, University of Verona, 37124 Verona, Italy; erika.rigotti@aovr.veneto.it (E.R.); giorgio.piacentini@univr.it (G.P.); 3 Research and Innovation Unit, University Hospital of Parma, 43126 Parma, Italy; ccaminiti@ao.pr.it; 4 Infectious Diseases Unit, IRCCS Giannina Gaslini, 16147 Genoa, Italy; eliocastagnola@gaslini.org; 5 Paediatric and Infectious Disease Unit, Academic Department of Pediatrics, IRCCS Bambino Gesù Children’s Hospital, 00165 Rome, Italy; laura.lancella@opbg.net; 6 Pediatric Infectious Disease Unit, Meyer Children’s Hospital, 50139 Florence, Italy; elisabetta.venturini@meyer.it; 7 Unit of Immune and Infectious Diseases, IRCCS Bambino Gesu’ Children’s Hospital, 00165 Rome, Italy; maia.deluca@opbg.net; 8 Institute for Biomedical Research and Innovation, National Research Council, 90146 Palermo, Italy; stefania.lagrutta@irib.cnr.it; 9 Paediatric Surgery, IRCCS Azienda Ospedaliera-Universitaria di Bologna, 40138 Bologna, Italy; mario.lima@unibo.it; 10 Division of Anesthesia, Analgesia, and Intensive Care, Department of Surgical and Biomedical Sciences, University of Perugia, 06129 Perugia, Italy; simonettatesoro@gmail.com; 11 Neonatal Intensive Care Unit, Department of Women’s and Children’s Health, Maggiore Hospital, 40133 Bologna, Italy; matilde.ciccia@ausl.bologna.it; 12 Department of Translational Medical Science, Section of Pediatrics, University of Naples “Federico II”, 80131 Naples, Italy; staiano@unina.it; 13 Università degli Studi di Milano, 20122 Milan, Italy; nicola.principi@unimi.it

**Keywords:** cystoscopy, hypospadias, kidney transplantation, nephrolithiasis, RAND/UCLA method, surgical antimicrobial prophylaxis, urology

## Abstract

The main aim of surgical antimicrobial prophylaxis (SAP) in urologic procedures is to prevent bacteraemia, surgical site infections (SSIs), and postoperative urinary tract infections (ppUTIs). Guidelines for SAP in paediatric urology are lacking. Only some aspects of this complex topic have been studied, and the use of antibiotic prophylaxis prior to surgical procedures seems to be more often linked to institutional schools of thought or experts’ opinions than to rules dictated by studies demonstrating the most correct and preferred management. Therefore, the aim of this Consensus document realized using the RAND/UCLA appropriateness method is to provide clinicians with a series of recommendations on SAP for the prevention of bacteraemia, SSIs, and ppUTIs after urologic imaging and surgical procedures in paediatric patients. Despite the few available studies, experts agree on some basilar concepts related to SAP for urologic procedures in paediatric patients. Before any urological procedure is conducted, UTI must be excluded. Clean procedures do not require SAP, with the exception of prosthetic device implantation and groin and perineal incisions where the SSI risk may be increased. In contrast, SAP is needed in clean-contaminated procedures. Studies have also suggested the safety of eliminating SAP in paediatric hernia repair and orchiopexy. To limit the emergence of resistance, every effort to reduce and rationalize antibiotic consumption for SAP must be made. Increased use of antibiotic stewardship can be greatly effective in this regard.

## 1. Introduction

The main aim of surgical antimicrobial prophylaxis (SAP) in urologic procedures is to prevent bacteraemia, surgical site infections (SSIs), and postoperative urinary tract infections (ppUTIs) [1]. Studies carried out in adults have tried to identify the most relevant risk factors for the development of urologic surgery-related infections, the clinical conditions in which antibiotics could be recommended, and finally, which antibiotics are appropriate for each clinical condition and when and how they should be prescribed [2,3]. Unfortunately, mainly due to the relatively low number of appropriately conducted randomized controlled clinical trials, national guidelines developed by local experts have frequently differed. For several urological procedures, different conclusions have been drawn, making it very difficult to decide which could be the most effective and safe antibiotic prescription. This explains why most of the studies carried out to evaluate how antibiotics are used to prevent infections in patients undergoing urological procedures have shown substantial variations in practice patterns among surveyed urologists [4,5,6].

Studies on SAP in urological procedures are even less numerous in children than in adults. Guidelines for antibiotic prophylaxis in paediatric urological surgery are lacking. Only some aspects of this complex topic have been studied, and the use of antibiotic prophylaxis prior to surgical procedures seems to be more often linked to institutional schools of thought or experts’ opinions than to rules dictated by studies demonstrating the most correct and preferred management. Moreover, as children are not small adults and the genitourinary system significantly changes from infancy to adolescence, adult guidelines for the prevention of infectious complications after urologic surgery are not directly applicable to the paediatric population. Therefore, the aim of this Consensus document is to provide clinicians with a series of recommendations on prophylaxis for the prevention of bacteraemia, SSIs, and ppUTIs after urologic imaging and surgical procedures in paediatric patients (0–18 years old). The recommendations are based on a careful review of the available scientific evidence and on the evaluation of a multidisciplinary group of experts regarding the choice of the molecule, dosage, time, and duration of administration for the most common urological surgical procedures.

## 2. Methods

### 2.1. RAND/UCLA Appropriateness Method

The Consensus document was realized using the Research and Development Corporation (RAND) and the University of California—Los Angeles (UCLA) appropriateness method. The RAND/UCLA appropriateness method consists of the appropriateness evaluation of diagnostic and therapeutic procedures with sub-optimal scientific evidence, by a panel of experts [7]. According to the RAND method, a procedure is defined as “appropriate” if the expected benefits outweigh the expected negative consequences, with a wide margin that justifies it, regardless of the costs. On the contrary, a procedure whose expected risks outweighs the expected benefits is considered as “inappropriate”. According to the RAND definition, the expert who makes an appropriateness/inappropriateness judgment must consider the clinical benefits and not be influenced by economic considerations. Therefore, the appropriateness accounts for the evaluation of the risk/benefit ratio of a list of management and therapeutic procedures [8]. For a heterogeneous topic such as SAP on which randomized controlled trials in paediatrics are lacking, the application of methods aiming to increase the homogeneity of behaviours by neonatologists, infectious diseases specialists, paediatric surgeons, and anaesthetists appeared useful and appropriate. For this reason, also considering recent positive experience in paediatrics [9,10,11], the RAND/UCLA approach was chosen instead of GRADE methodology. Through the RAND method, the participants discussed different clinical scenarios and elaborated statements on the basis of the literature and their clinical experience. The group of experts did not consider it appropriate to combine the GRADE method with the RAND/UCLA approach because the absence of randomized studies represents a bias in defining the strength of the recommendations and in representing a consensus reached for real-life.

### 2.2. Recruitment of Panellists

A multidisciplinary group of experts belonging to the main Italian scientific societies dealing with anti-infective therapy in paediatric age was selected. The following Scientific Societies were involved: Italian Society of Paediatrics (SIP), Italian Society of Neonatology (SIN), Italian Society of Paediatric Infectious Diseases (SITIP), Italian Society of Infectious and Tropical Diseases (SIMIT), Italian Society of Paediatric Surgery (SICP), Italian Society of Microbiology (SIM), Italian Society of Pharmacology (SIF), Italian Society of Anaesthesia and Neonatal and Paediatric Resuscitation (SARNEPI), Italian Society of Childhood Respiratory Diseases (SIMRI). The panel of experts was made up of 52 medical doctors with at least a 5 years of experience: paediatricians (n = 20), neonatologists (n = 6), infectious diseases specialists (n = 5), paediatric surgeons (n = 5), anaesthetists (n = 8), pharmacologists (n = 5), and microbiologists (n = 3).

### 2.3. Generation of Scenarios

Initially, a literature search was performed with a selection of documents including randomized studies, systematic reviews of the literature, meta-analyses and guidelines on peri-operative prophylaxis for the prevention of SSI in neonatal and paediatric urologic surgery. The literature search was carried out on the PubMed Database, with a choice of articles in English only, published from the year 2000 until 2020. The following key terms were used: “antimicrobial prophylaxis” OR “antibiotic prophylaxis” AND “urologic surgery” OR “bladder” OR “vescicoureteral reflux” OR “epispadias” OR “ureteropelvic junction obstruction” OR “ureterovescical junction obstruction” OR “urethrography” OR “urodynamics” OR “cystourethrography” OR “cystography” OR “cystoscopy” OR “circumcision” OR “penile excision” OR “penile repair” OR “inguinal hernia” OR “orchidopexy” OR “urethromeatoplasty” OR “scrotal” OR “nephrectomy” OR “cystectomy” OR “kidney transplantation” OR “nephrolithiasis” OR “lithotripsy” OR “ureteroscopy” OR “nephtolithotomy” OR “hypospadias” AND “neonate” OR “newborn” OR “paediatric” OR “pediatric” OR “children” OR “adolescent”. Subsequently, using the Patient/Problem/Population-Intervention-Comparison/Control/Comparator-Outcome (PICO) model, a questionnaire was created on SAP in neonatal and paediatric urology relating to different procedures, which were divided into 7 clinical scenarios. Before administration, it was tested twice with a one-week interval to a convenience sample of 4 paediatricians, 2 neonatologists, 1 infectious diseases specialist, 1 paediatric surgeon, 1 anaesthetist, 1 pharmacologist, and 1 microbiologist. Then, 26 out of 52 experts were selected by the Scientific Societies for answering, and the questionnaire was administered to 11 paediatricians, 3 neonatologists, 2 infectious diseases specialists, 3 paediatric surgeons, 4 anaesthetists, 2 pharmacologists, and 1 microbiologist.

### 2.4. Two-Round Consensus Process

On the basis of the 7 scenarios, the questionnaire was submitted to the experts on the “REDCap” online platform. Each question included the clinical scenario and possible answers were whether or not SAP was recommended for the scenario and, in case of its recommendation, a list with all the antibiotics available on the EU market so that the expert could select the antibiotics that he/she considered as first choice. The selected bibliographic material was made available to all panel members, who were instructed on how to fill in the panel. The experts answered anonymously to the questionnaire and their judgment was expressed on a 1–9 scale, where “1” was considered definitely inappropriate, “5” was considered uncertain, and 9 was considered definitely appropriate. Intermediate values corresponded to different modulations of the judgment of inappropriateness (“2” and “3”), uncertainty (from “4” to “6”), and appropriateness (“7” and “8”), respectively. In evaluating each indication, each expert referred both to his/her own experience and clinical judgment and to the available scientific evidence. A free space was provided for any annotation or comment.

The first round of the questionnaire was blind to other panel members. Multiple participation was not permitted by the platform, that guaranteed also the confidentiality and anonymity of the answers. Results of the survey were discussed in a collegial meeting with all the 26 experts who answered the questionnaire to find an agreement and reduce eventual disagreement. Clarifications, adaptations, and refinements of the indications and appropriateness ratings were made. A total of 7 recommendations were developed (i.e., one for each scenario). All the 52 participants were asked to approve the recommendations in a second round during the following four weeks. After the revision of the manuscript on the basis of comments received by the reviewers, an additional discussion by e-mail was performed and, considering supporting evidence, the 52 participants were asked to approve the recommendations in a further round during the following week.

## 3. Results

### 3.1. SCENARIO #1. Imaging Procedures Involving the Urinary Tract

Imaging procedures such as retrograde urethrography (RUG), urodynamics (UDS), voiding cystourethrography (VCUG), and radionuclide cystography (RNC) require catheterization and retrograde instillation of contrast. They are believed to pose an inherent risk of ppUTI. Given the high incidence of urologic anatomic abnormalities in children undergoing one-time urinary catheterization procedures, the development of ppUTIs may result in severe clinical problems, including death from urosepsis [12]. This explains why SAP is commonly prescribed to children undergoing RUG, UDS, VCUG, and RNC [13].

However, studies carried out in children undergoing imaging procedures requiring catheterization have shown that the risk of ppUTI is generally low, although with some exceptions [14,15,16,17,18,19,20]. In children undergoing RUG or UDS, ppUTI was documented in 2.1% and 0–4.8% of cases, respectively. Larger variations were evidenced in studies enrolling children with VCUG and RNC, among whom ppUTI was diagnosed in up to 42% of children [14,15,16,17,18,19,20]. The highest values were reported in the oldest studies, while in the most recent evaluations incidence rates of ppUTI were found in the range already reported for the other imaging procedures with catheterization. Examples in this regard are the studies by Johnson et al. [18] and Marzuillo et al. [20]. Johnson et al. retrospectively examined 1203 cystograms (1157 VCUGs and 46 RNCs) among which 33% were carried out in children who were receiving antibiotic prophylaxis. PpUTI was detected in only 12 children (1%), of whom 7 were receiving prophylaxis [18]. Among 216 children undergoing cystoscopy, Marzuillo et al. retrospectively evaluated 216 non-toilet-trained children not assuming antibiotics when undergoing cystography and found that only 5 (6.6%)% developed a UTI. [20]. Differences in criteria used for UTI diagnosis and characteristics of the study population can explain variations in ppUTI detection. Values were higher when, together with symptomatic ppUTIs, clinically irrelevant asymptomatic ppUTIs were also considered [17,18,20]. Moreover, the frequency was higher when the studied paediatric population included a great number of patients with pre-existing urologic abnormalities, especially high-grade reflux, all conditions that are well-known risk factors for ppUTI development. In the study by Marzuillo et al., all children without vescico-reteral reflux (VUR) or with low-grade reflux did not develop ppUTI [20]. In contrast, among children with high-grade VUR, 6% had ppUTIs [20]. However, administration of antibiotics does not exclude ppUTI development, at least in children with urologic abnormalities. Sinha et al. treated children undergoing VCUG with antibiotics (cephalexin if <6 months or trimethoprim/sulfamethoxazole if >6 months old) because of various urological problems and reported that children with an abnormal preprocedure renal ultrasound were at increased risk of ppUTI (odds ratio [OR] 9.51, 95% confidence interval [CI] 1.43 to 63.4) [19]. Although available evidence does not allow us to draw firm conclusions on the risk of ppUTI after one-time urinary catheterization procedures and the efficacy of antibiotic prophylaxis, it may be suggested that antibiotics might be given to children with strongly suspected or already proven urinary abnormalities when they undergo RUG, UDS, VCUG, and RNC. Unfortunately, it is not established which are the most effective and safe drugs, when and how they must be given. Considering that *Enterococci* and Gram-negative bacilli are the most likely infecting agents, in accordance with the recommendations of the American Urological Association [21], the combination trimethoprim/sulfamethoxazole (2 mg/kg of trimethoprim component p.o. in patients >6 weeks of age) or amoxicillin/clavulanic acid (50 mg/kg of the amoxicillin component p.o.) or gentamicin (2.5 mg/kg i.v./i.m.) immediately prior to intervention can be recommended in absence of bladder and bowel dysfunction. The absence of reliable sources does not allow to verify the relevance of continuing prophylaxis during the 24 h post-procedure.

**Recommendation 1**. SAP with trimethoprim/sulfamethoxazole (2 mg/kg of trimethoprim component p.o. in patients >6 weeks of age) or amoxicillin/clavulanic acid (50 mg/kg of the amoxicillin component) or gentamicin (2.5 mg/kg i.v./i.m.) immediately prior to intervention is recommended for children with strongly suspected or already proven urinary abnormalities when they undergo RUG, UDS, VCUG, and RNC.

### 3.2. SCENARIO #2. Cystoscopy and Other Endoscopic Procedures

Cystoscopy, ureteroscopy, and all the other endoscopic procedures are associated with a potential risk of ppUTI that is reported in up to 10% of patients [18]. Official recommendations for adults consider pre-procedure administration only in subjects with risk factors, including urological malformations and previous history of UTI, particularly in recurrent cases [22]. This is because studies comparing ppUTIs in otherwise healthy patients have shown that the absolute incidence of ppUTIs in these procedures is low and that antimicrobials do not reduce the risk. In children with urological malformations and previous history of UTI, the combination trimethoprim/sulfamethoxazole (2 mg/kg of trimethoprim component p.o. in patients >6 weeks of age) or amoxicillin/clavulanic acid (50 mg/kg of the amoxicillin component) or gentamicin (2.5 mg/kg i.v./i.m.) are recommended before endoscopic procedures.

**Recommendation 2**. SAP with trimethoprim/sulfamethoxazole (2 mg/kg of trimethoprim component p.o. in patients >6 weeks of age) or amoxicillin/clavulanic/acid (50 mg/kg of the amoxicillin component) or gentamicin (2.5 mg/kg i.v./i.m.) is recommended in children with urological malformations or a previous history of recurrent UTI before cystoscopy, ureteroscopy, and all other endoscopic procedures.

### 3.3. SCENARIO #3. Clean Urological Procedures

Some surgical procedures are considered clean procedures, as the operative wounds have no inflammation and do not enter the respiratory, gastrointestinal, and genitourinary tracts [23]. In adults, it has been shown that in patients undergoing these procedures, the risk of bacterial superimposed infection is low, and that SAP does not reduce this risk. SAP is not recommended by several scientific societies, with the exception of prosthetic device implantation and groin and perineal incisions, where the SSI risk may be increased [21,24]. Studies in children seem to confirm these findings. A retrospective cross-sectional evaluation of all paediatric clean surgical procedures performed in a group of 178 patients (mean age: 8.19 years) during a two-year period was conducted by Syed et al. [25]. Only children who had not received antibiotics at least two weeks prior to the procedure were enrolled. Paediatric patients with an increased risk of infection, such as those with immunodeficiency, congenital heart disease, ventriculoperitoneal shunt, and nephrotic syndrome, were excluded. The development of SSIs was monitored for two to four weeks after surgery. Only one case of postoperative SSI was reported, accounting for a prevalence of 0.56% [25].

Among the genitourinary surgery procedures, circumcision/circumcision revisions, penile skin bridge excision, chordee repair, penile torsion repair, inguinal hernia repair, scrotal and inguinal orchidopexy, urethromeatoplasty, and scrotal procedures are the most common clean procedures performed in children [25]. A study specifically evaluating the risk of postsurgical infections in 885 children undergoing these procedures showed that this risk was 0.33% in children without SAP and 0.35% in those given antibiotics, confirming that SAP is not needed in clean urologic surgery [26].

Further data highlighting these conclusions have been collected in studies enrolling children undergoing selected urological clean procedures. Regarding orchiopexy, alone or with herniorrhaphy, a retrospective study enrolling 71,767 children (mean age, 4.6 years) undergoing surgery in an ambulatory or observation setting showed that the development of SSI among those who did not receive SAP (23,986, 33.5%) was evidenced in only 0.1% of the cases; the same value was calculated in those given antibiotics [27]. Moreover, SAP did not influence the rates of hospital readmission or any repeat hospital encounter within 30 days of surgery. In contrast, children receiving SAP had 1.2 times the odds of a perioperative allergic reaction compared with those who did not receive SAP (*p* = 0.005). Unfortunately, the type and duration of antibiotic therapy were not reported [27].

Regarding circumcision, a retrospective study of 84,226 males (median age, 2.2 years) who underwent this procedure, among whom 8944 (10.6%) received SAP, did not show any associations between SAP and SSI prevention (0.1% vs. 0.2%; *p* = 0.5), penile reoperation (0.01% vs. 0.04%; *p* = 0.4), or hospital visit (5.5% vs. 5.5%; *p* = 0.8). Patients with SAP had 1.5 times the odds of an allergic reaction (OR 1.5; 95% CI, 1.3–1.7; *p* < 0.0001) compared with those who did not; in detail, children younger than 5 years old were significantly more likely to have an allergic reaction (<1 year old: OR 32.9, ≥1 and <5 years old: OR 28.6, *p* < 0.0001) than adolescents. Moreover, those who received SAP were also 1.2 times more likely to get a hospital visit (OR 1.2; 95% CI, 1.1–1.3; *p* = 0.0021) than those without SAP [28]. Considering the available data, there is strong evidence that children undergoing clean urological procedures do not need antibiotic prophylaxis. The risk of antibiotic-related allergic reactions strongly supports this statement.

**Recommendation 3**. In children undergoing clean urological procedures (i.e., circumcision/circumcision revisions, penile skin bridge excision, chordee repair, penile torsion repair, inguinal hernia repair, scrotal and inguinal orchidopexy, urethromeatoplasty, and scrotal procedures), SAP is not recommended.

### 3.4. SCENARIO #4. Clean-Contaminated Urological Procedures

Clean-contaminated procedures include those entering respiratory, gastrointestinal, or genitourinary systems [23]. In urology, clean-contaminated procedures include any opening into the genitourinary tract, as well as nephrectomy, cystectomy, endoscopy, and vaginal procedures [23].

Open interventions require systematic SAP, as the risk reduction of a serious surgical site infection or systemic infection exceeds the anticipated risks of increasing antimicrobial resistance and other adverse events. Recommendations for adults differ according to the type of surgical procedure, although a single preoperative dose of the drug(s) administered 1–2 h before surgical incision or start of the procedure is considered adequate to prevent SSI. The American Urological Association suggests, as first choice, the use of cefazolin or trimethoprim/sulfamethoxazole if the procedure involves controlled entry into the urinary tract and cefazolin plus metronidazole when large bowel is involved [21]. This because in the first case the most common potential pathogens are the common urinary tract organisms such as *Escherichia coli*, *Proteus* spp, *Klebsiella* spp., whereas in the second case also *Enterococci* and anaerobes can be the cause of the infection. Limited data are available for children. Health Improvement Scotland (HIS) and the Scottish Intercollegiate Guidelines Network (SIGN) recommends that when surgery involves the urinary system alone, gentamicin 2.5 mg/kg i.v./i.m. pre-surgically and postoperative trimethoprim/sulfamethoxazole (2 mg/kg of trimethoprim component p.o. in patients >6 weeks of age) at night until stent removal are suggested [29]. In the case of previous trimethoprim resistance, amoxicillin/clavulanic acid once daily at night is recommended. When a urological procedure results in entry into the bowel, cefotaxime 50 mg/kg i.v. and metronidazole 7.5 mg/kg i.v. pre-surgically and every 4 h during surgery are recommended [29].

**Recommendation 4.** In cases of clean-contaminated urological procedures in paediatric patients (i.e., any opening into the genitourinary tract, nephrectomy, cystectomy, endoscopy, and vaginal procedures), gentamicin 2.5 mg/kg i.v./i.m. pre-surgically and post-operative trimethoprim/sulfamethoxazole (2 mg/kg of trimethoprim component p.o. in patients >6 weeks of age) at night until stent removal are recommended. In the presence of trimethoprim resistance, amoxicillin/clavulanic acid (50 mg/kg of the amoxicillin component) once daily is recommended until stent removal. When urological procedures result in entry into the bowel, cefotaxime 50 mg/kg i.v. and metronidazole 7.5 mg/kg i.v. pre-surgically and every 4 h during surgery are recommended.

### 3.5. SCENARIO #5. Kidney Transplantation

Studies in the adult population revealed that SSI and ppUTI might occur in up to 30% of patients [30]. Including the asymptomatic bacteriuria, the frequency of complicated patients would significantly raise. However, it’s more likely that only the symptomatic febrile patients with ppUTIs are prone to acute graft loss, renal scarring, and increased mortality. Female sex, older age, reflux kidney disease, and days of bladder catheterization have been shown to be relevant risk factors for complications in adults [30]. In SSIs, Gram-positive bacteria are the most common and the proportion of multi-drug-resistant strains is increasing [30]. In children and adolescents, risk factors for posttransplant UTI are urological causes of renal failure, indwelling catheters and stents, and history of pretransplant UTI [30]. To reduce infection risk, SAP is generally used in patients undergoing kidney transplantation, although its real relevance is not precisely defined. A retrospective comparison of peri-operative intravenous cefazolin prophylaxis compared to no antibiotic showed no difference in infectious complication rates in the first month after surgery [31]. Regarding antibiotic administration, a multicentre, prospective randomized controlled trial showed no difference in efficacy between a single dose broad spectrum antibiotic at induction of anaesthesia and the administration of antibiotic 12 hourly for 3–5 days [32]. This explain why the use of a single-dose, rather than multi-dose, peri-operative prophylactic antibiotics in routine renal transplant recipients is generally recommended. In adults, cefazolin (2 g i.v. within the one-hour time frame and further doses every four hours throughout surgery) is recommended by both American [33] and European institutions [34]. No definitive recommendations have been developed for children and recommendations reported for adults may be used. However, some experts suggest that cefazolin i.v. (50 mg/kg; max 2 g) should be replaced by a single dose of piperacillin/tazobactam i.v. 100 mg/kg (as piperacillin) over 30 min before incision (max 4 g piperacillin component) or, in case of delayed or immediate sensitivity with meropenem i.v. (20 mg/kg/dose; max 1 g) or aztreonam i.v. (30 mg/kg/dose; max 2 g) plus vancomycin i.v. (10 mg/kg; max 500 mg), respectively [35]. This to overcome the increasing incidence of UTIs caused by extended-spectrum β-lactamase (ESBL)-producing *Enterobacteriaceae* [36]. In the patient hospitalized for >2 weeks, it is recommended to carry out screening by nasal swab for the search for colonization by *Staphylococcus aureus* (both methicillin-susceptible and methicillin-resistant).

**Recommendation 5.** In the case of kidney transplantation in paediatric patients, cefazolin 50 mg/kg i.v. (max 2 g) as a single dose within 30 min before incision is recommended as SAP. In those geographic areas where incidence of extended-spectrum β-lactamase (ESBL)-producing *Enterobacteriaceae* is high, the combination piperacillin/tazobactam i.v. 100 mg/kg (as piperacillin; max 4 g) with the same timing is recommended.

### 3.6. SCENARIO #6. Stone Therapy

Options for surgical removal of nephrolithiasis in patients with a normal urinary system include extracorporeal shock wave lithotripsy (ESWL), ureteroscopy (UTS), and percutaneous nephrolithotomy (PCNL) [37,38]. Open or laparoscopic stone removal should be reserved for patients with anatomical abnormalities [37,38]. In adults, a recent analysis of published studies has shown that ESWL is associated with the development of UTIs and fever in 4.2% and 3.4% of cases and that SAP does not reduce either the risk of ppUTIs (relative risk [RR], 0.76; 95% CI, 0.39–1.48; *p* = 0.42) or the incidence of fever (RR, 0.26; 95% CI, 0.06–1.10; *p* = 0.07) [38]. Consequently, preoperative SAP is not recommended apart from patients with a large stone burden, associated pyuria, history of pyelonephritis, and adjunctive operative procedures, as these subjects are considered at the highest risk of infectious complications. In contrast, in non-ESWL stone manipulation procedures, SAP is recommended, as it can frequently cause postprocedural UTI and fever (33.4% and 21.7%, respectively). SAP is effective in reducing the UTI incidence (RR, 0.30; 95% CI, 0.15–0.58; *p* < 0.001), although the effect on fever (RR, 0.38; 95% CI, 0.12–1.21; *p* = 0.10) remains undefined [38]. When SAP is required, cefazolin or trimethoprim/sulfamethoxazole are the agents of choice [38]. Use of second or third generations cephalosporins should be considered where risk of antibiotic resistance of the most common urinary pathogens is high.

Studies on infectious complications in children undergoing stone therapy are lacking. Recommendations reported for adults could also be followed for children. Practically, SAP can be recommended in children undergoing ESWL if they have a history of previous UTI, large stone burden and anatomical abnormalities and in all children with non-ESWL stone manipulation. As *Staphylococcus aureus*, coagulase-negative *Staphylococcus* and Gram-negative rods are the most common infecting pathogens, cefazolin 30 mg/kg i.v. could be considered the drug of choice and should be administered only before the procedure. Trimethoprim/sulfamethoxazole (2 mg/kg of trimethoprim component p.o. in patients >6 weeks of age) can be an alternative

**Recommendation 6.** SAP with cefazolin (30 mg/kg i.v.; max 2 g) or trimethoprim/sulfamethoxazole (2 mg/kg of trimethoprim component p.o. in patients >6 weeks of age) only before the procedure (within 30 min before incision) is recommended in children undergoing ESWL if they have a history of previous UTI, large stone burden and anatomical abnormalities and in all children with non-ESWL stone manipulation.

### 3.7. SCENARIO #7. Hypospadias Repair

Hypospadias repair is considered a clean-contaminated procedure that is generally accompanied by the placement of a urethral tube for urinary drainage after surgery to reduce the risk of complications, such as urethrocutaneous fistula [39,40,41]. The surgical procedure and urethral tube placement are considered potential causes of both SSI and UTI, and prescription of antibiotics from the time of surgery through postoperative catheter removal is a common practice among both adult [42] and paediatric urologists [43]. However, assessment of SSI and ppUTI incidence rates after hypospadias repair by means of cohorts or randomized controlled trials has shown that, contrary to physicians’ belief, the risk of infectious complications after this kind of surgery is very low, and the administration of antimicrobial drugs does not lead to substantial benefit. Regarding children, a recent study enrolling 441 subjects compared the use of preoperative prophylaxis, postoperative prophylaxis, combined pre- and postoperative prophylaxis and no prophylaxis on the incidence of both SSI and postsurgical complications after hypospadias repair. The results showed that infectious problems even in untreated children are rare and that treatment, regardless of type, does not modify the risk of infections [44]. Similar findings were previously reported by Smith et al. [45] and by Chua et al. [46]. Smith et al. studied only the role of preoperative prophylaxis, showing that this has no role because of the very poor risk of infectious development in untreated children [45]. Chua et al. performed a meta-analysis of seven studies published before March 2018 regarding postoperative prophylaxis and did not find any differences in outcomes of composite overall post-hypospadias repair complications (RR, 0.93; 95% CI, 0.45, 1.93) between children with and children without antibiotics [46]. No differences were found even when UTI and SSI were considered separately (RR, 1.28, 95% CI 0.49–3.35, and RR, 1.01, 95% CI 0.48–2.12, respectively). Only asymptomatic bacteriuria was found to be significantly higher among children without postoperative prophylactic antibiotics (RR, 4.01; 95% CI 1.11–14.54) [46]. Unfortunately, most of the cited studies have significant methodological limitations that do not allow us to draw firm conclusions. This explains why the need for pre- and/or postoperative prophylactic antibiotic administration remains debated, and some experts continue to recommend antibiotic prophylactic administration. A single preoperative dose of a first or a second cephalosporin is generally recommended for adults, with gentamicin as second choice [33]. Available data regarding children are few and conflicting, leading to recommendations based only on personal opinion and not reliable data. The Royal Hospital for Children, Glasgow [29] does not recommend prophylaxis, whereas the Children’s Health Queensland Hospital and Health Service [35] suggests the use of cefazolin i.v. before incision, followed by oral trimethoprim/sulfamethoxazole until catheter is removed. Gentamicin 5 mg/kg i.v. (infuse over 30 min) can be used as a cefazolin substitution.

**Recommendation 7.** In hypospadias repair, it is recommended to administer cefazolin i.v. (30 mg/kg; max 2 g) within 30 min before incision followed by oral trimethoprim/sulfamethoxazole (2 mg/kg of trimethoprim component p.o. in patients >6 weeks of age) Until the catheter is removed.

## 4. Discussion

Table 1 summarizes the experts’ recommendations on SAP for urologic procedures in paediatric patients.

Preoperative UTI, especially if recurrent, is a major risk factor for SSI and ppUTI [1]. Before any urological procedure is conducted, UTI must be excluded. Clean procedures (i.e., uninfected, no inflammation, closed primarily without entrance into the gastrointestinal or genitourinary tracts) do not require prophylaxis, with the exception of prosthetic device implantation and groin and perineal incisions where the surgical site infection risk may be increased [24,25,26,27,28]. In contrast, prophylaxis is needed in clean-contaminated procedures [30,37,38]. Subjects with urinary tract malformations with or without obstruction, lithiasis, and indwelling or externalized catheters frequently develop postsurgical infections and must be given antibiotics for prophylaxis. Studies have also suggested the safety of eliminating SAP in paediatric hernia repair and orchiopexy [27]; however, the relatively small cohorts of patients and the rare incidence of postoperative surgical site infections require a large patient cohort to determine if a true benefit (or detriment) exists.

The lack of evidence-based guidelines regarding SAP for urological procedures in paediatric patients has led to significant variability in the use of antibiotics. Interestingly, Chan et al. showed that clinicians using SAP for clean procedures had a higher likelihood of using SAP for clean-contaminated procedures [47]. In contrast, those not using SAP for clean-contaminated procedures had a higher likelihood of not using SAP for clean procedures [47]. Our recommendation put forward for trimethoprim/sulfamethoxazole in infants >6 weeks of age. Because of short exposure duration, the risk of kernicterus is limited, and this recommendation could avoid increasing further antimicrobial resistance to beta-lactams [48].

Through the RAND method, in our study the participants discussed the statements derived from the guidelines and the agreement was reached in the recommendations for SAP in urologic procedures. It should be noted that the participants in the project came from different clinical contexts, i.e., they were paediatricians, neonatologists, infectious diseases specialists, paediatric surgeons, anaesthetists, pharmacologists and microbiologists. For this reason, the results achieved demonstrate the usefulness of the RAND method for the selection of good practices and constitute the basis of an evidence-based approach. The findings obtained can establish the basis for educational interventions that aim to optimize the use of antibiotics in urologic paediatric patients. Limitations of the study included that this was an opinion-based survey to base recommendations and the agreement was reached on a collegial meeting. On the other hand, the lack of paediatric studies on urologic procedures did not permit to use the GRADE methodology and the complexity of the topic required an online face-to-face meeting with all the participants. However, the RAND method did not permit to define a hierarchy of antibiotics’ administration and not using the GRADE method may affect the quality of these recommendations.

## 5. Conclusions

The indications for antibiotic prophylaxis in paediatric urological procedures are often borrowed from adult experience or from professional expertise, but there are no univocal indications. In recent years, with the increase in UTI from antibiotic-resistant bacteria [49,50], the choice of molecules to be used for antibiotic prophylaxis is even more complex. This highlights the concept that continuous monitoring of the microbiological characteristics of UTIs and updating of the recommendations for antibiotic choice are needed. To limit the emergence of resistance, every effort to reduce and rationalize antibiotic consumption for SAP must be made. Increased use of antibiotic stewardship [51] can be greatly effective in this regard. This manuscript, made possible by the multidisciplinary contribution of experts belonging to the most important Italian scientific societies, represents, in our opinion, the most up-to-date and comprehensive collection of recommendations on SAP during urologic procedures in paediatric patients. Our panel of experts thinks that, in the face of extremely heterogeneous prescriptions in real life, characterized by an excessive and often inappropriate use of antibiotics in SAP, our document represents a balanced and shared text, derived from an extensive discussion also following the comments received from reviewers, which can be extraordinarily beneficial for the patient and, more generally, for the health system. As soon as our Consensus document will be implemented by the Italian Scientific Societies, it will be interesting to analyse its clinical and economic impact in our geographical context.

## Figures and Tables

**Table 1:** Surgical antimicrobial prophylaxis (SAP) for urologic procedures in paediatric patients.

Type of Urologic Procedure	Recommendation
Imaging procedures such as retrograde urethrography, urodynamics, voiding cystourethrography, and radionuclide cystography that require catheterization and retrograde instillation of contrast	SAP with trimethoprim/sulfamethoxazole (2 mg/kg of trimethoprim component p.o. in patients >6 weeks of age) or amoxicillin/clavulanic acid (50 mg/kg of the amoxicillin component) or gentamicin (2.5 mg/kg i.v./i.m.) immediately prior to intervention is recommended to children with strongly suspected or already proven urinary abnormalities.
Cystoscopy, ureteroscopy, and all other endoscopic procedures.	SAP with trimethoprim/sulfamethoxazole (2 mg/kg of trimethoprim component p.o. in patients >6 weeks of age) or amoxicillin/clavulanic/acid (50 mg/kg of the amoxicillin component) or gentamicin (2.5 mg/kg i.v./i.m.) is recommended in children with urological malformations or a previous history of recurrent UTI.
Clean urological procedures (i.e., circumcision/circumcision revisions, penile skin bridge excision, chordee repair, penile torsion repair, inguinal hernia repair, scrotal and inguinal orchidopexy, urethromeatoplasty, and scrotal procedures)	SAP is not recommended.
Clean-contaminated urological procedures in paediatric patients (i.e., any opening into the genitourinary tract, nephrectomy, cystectomy, endoscopy, and vaginal procedures)	Gentamicin (2.5 mg/kg i.v./i.m.) pre-surgically and post-operative trimethoprim/sulfamethoxazole (2 mg/kg of trimethoprim component p.o. in patients >6 weeks of age) at night until stent removal are recommended. In the presence of trimethoprim resistance, amoxicillin/clavulanic acid (50 mg/kg of the amoxicillin component) once daily is recommended until stent removal. When urological procedures result in entry into the bowel, cefotaxime (50 mg/kg i.v.) and metronidazole (7.5 mg/kg i.v.) pre-surgically and every 4 h during surgery are recommended.
Kidney transplantation	SAP with cefazolin (30 mg/kg i.v.; max 2 g) as a single dose within 30 min before incision is recommended. In those geographic areas where incidence of extended-spectrum β-lactamase (ESBL)-producing *Enterobacteriaceae* is high piperacillin/tazobactam i.v. 100 mg/kg (as piperacillin; max 4 g) is recommended.
Stone therapy	SAP with cefazolin (30 mg/kg i.v.; max 2 g) or trimethoprim/sulfamethoxazole (2 mg/kg of trimethoprim component p.o. in patients >6 weeks of age) only before the procedure (within 30 min before incision) is recommended in children undergoing ESWL if they have a history of previous UTI, large stone burden and anatomical abnormalities and in all children with non-ESWL stone manipulation.
Hypospadias repair	SAP with cefazolin (30 mg/kg i.v.; max 2 g) within 30 min before incision followed by oral trimethoprim/sulfamethoxazole (2 mg/kg of trimethoprim component p.o. in patients >6 weeks of age) until catheter is removed is recommended.

ESWL, extracorporeal shock wave lithotripsy; UTI, urinary tract infection.

## Data Availability

All the data are included in the manuscript.
